# The bio-distribution of the antidepressant clomipramine is modulated by chronic stress in mice: effects on behavior

**DOI:** 10.3389/fnbeh.2014.00445

**Published:** 2015-01-06

**Authors:** Georgia Balsevich, Christian Namendorf, Tamara Gerlach, Manfred Uhr, Mathias V. Schmidt

**Affiliations:** ^1^Department of Stress Neurobiology and Neurogenetics, Max Planck Institute of PsychiatryMunich, Germany; ^2^Department of Clinical Research, Max Planck Institute of PsychiatryMunich, Germany

**Keywords:** antidepressants, clomipramine, chronic social defeat stress, metabolic ratio, tissue distribution, mouse model

## Abstract

Major depression (MD) is one of the most common psychiatric disorders, severely affecting the quality of life of millions of people worldwide. Despite the availability of several classes of antidepressants, treatment efficacy is still very variable and many patients do not respond to the treatment. Clomipramine (CMI), a classical and widely used antidepressant, shows widespread interindividual variability of efficacy, while the environmental factors contributing to such variability remain unclear. We investigated whether chronic stress modulates the bio-distribution of CMI, and as a result the behavioral response to CMI treatment in a mouse model of chronic social defeat stress (CSDS). Our results show that stress exposure increased anxiety-like and depressive-like behaviors and altered the stress response. Chronic defeat stress furthermore significantly altered CMI bio-distribution. Interestingly, CMI bio-distribution highly correlated with anxiety-like and depressive-like behaviors only under basal conditions. Taken together, we provide first evidence demonstrating that chronic stress exposure modulates CMI bio-distribution and behavioral responses. This may contribute to CMI’s broad interindividual variability, and is especially relevant in clinical practice.

## Introduction

Major depression (MD) is a prevalent and debilitating disorder, affecting an estimated 350 million people worldwide (World Health Organisation, 2012). Despite the availability of several classes of antidepressants, symptom relief in the treatment of depression is often incomplete and highly variable between individuals (Labermaier et al., 2013). Currently, dose optimization for effective treatment of depression is achieved only by means of trial and error. Identification of early predictors of treatment response, including both genetic and environmental factors, is therefore an important step in optimizing the current treatment options for MD.

Stress is a major risk factor for depression, and dysregulation of the HPA axis as well as heightened stress reactivity are among the most consistent features in patients suffering from MD (Holsboer, 2000; Gallagher et al., 2007; Holsboer and Ising, 2010). Remission from MD is furthermore associated with the normalization of the HPA axis (Holsboer, 2000; Ising et al., 2007). However, there is a paucity of research exploring the effects of stress exposure on the therapeutic efficacy of various commonly used antidepressants.

Clomipramine (CMI), a tricyclic antidepressant, is commonly prescribed to treat depression, obsessive compulsive disorder, and panic disorders (Trimble, 1990). There is a broad interindividual variation in its efficacy, which is characteristic of many tricyclic antidepressants. The most well recognized processes contributing to interindividual variation in patient responses are pharmacokinetic in nature, influencing drug metabolism and drug partitioning (Shimoda et al., 1995; Vandel et al., 2004). Significant differences in the metabolic ratio have been described between patients who suffer from CMI side effects and those who present good tolerance to CMI. Importantly, the measurement of CMI and desmethylclomipramine (DCMI), an active metabolite, in the serum has been shown to improve dose optimization, and subsequently the therapeutic outcome to CMI treatment in patients (Mavissakalian et al., 1990; Noguchi et al., 1993; Marcourakis et al., 1999). For example, improved therapeutic outcome is associated with higher serum levels of CMI and DCMI as well as a higher metabolic ratio of CMI to DCMI (Szegedi et al., 1996). To-date, diverging pharmacokinetic factors and clinical responses to CMI observed amongst patients have been largely attributed to genetic factors (Basu et al., 2004; Kirchheiner et al., 2004). Identification of environmental factors that predict drug efficacy and/or patient response remains largely elusive despite the potential clinical implications. In particular, it is unknown whether chronic stress is able to actively modulate CMI metabolism or its distribution from the plasma to the brain, and potentially the therapeutic response to CMI treatment. Here, we combined a validated mouse model of chronic social defeat stress (CSDS) with CMI treatment in order to test whether stress is able to alter CMI metabolism, tissue partitioning, and treatment efficacy.

## Methods

### Animals and animal housing

Twelve-week old male C57BL/6 mice (Charles River Laboratories, Maastricht, Netherlands) were maintained under standard lab conditions (12:12 h light/dark cycle, controlled temperature (22 +/− 2°C) and humidity (55+/− 5%), and *ad libitum* access to food and water). Mice were singly-housed and acclimated to the room for 10 days before the experimental onset. The experiments were carried out in accordance with the European Communities’ Council Directive 2010/63/EU. All efforts were made to minimize animal suffering during the experiments. The protocols were approved by the committee for the Care and Use of Laboratory animals of the Government of Upper Bavaria, Germany.

### Experimental design

Mice were randomly assigned to 2 × 2 groups (control vehicle (*n* = 12), control CMI (*n* = 12), chronic stress vehicle (*n* = 12), and chronic stress CMI (*n* = 11)) counterbalanced by body weight. Clomipramine was administered orally through drinking water (0.12 mg/ml) from the first day of the stress procedure (day 1) until the animals were sacrificed (day 22). An open field (OF) test and forced swim test (FST) were performed on day 16 and day 18, respectively in order to assess CMI efficacy. We were mindful of the behavioral testing order, beginning with the least invasive test before testing more invasive assays (McIlwain et al., 2001; Paylor et al., 2006). Therefore the OF test was carried out before the FST and there was one full day of rest between the two tests. Body weight was measured daily and fluid intake was measured twice weekly throughout the entire experimental time-course. Behavioral testing and animal sacrifice always occurred in the morning at approximately the same time so that levels of metabolites measured at the time of sacrifice are representative of levels at the time of testing.

### Chronic social defeat stress procedure

The CSDS paradigm lasted for 21 days and was conducted as described previously (Wagner et al., 2011, 2012). Briefly, experimental mice were placed in the home cage of a dominant CD1 resident mouse. Interaction between the mice was permitted until the experimental mouse was attacked and defeated by the CD1 aggressor. Mice were subsequently separated by a wire mesh divider that prevented physical contact but maintained sensory contact for 24 h. Each day, for 21 days, the procedure was repeated with a different, unfamiliar CD1 aggressor mouse. Both control and stress animals were handled daily during the course of the stress procedure.

### Open field

Mice were placed in one corner of a 50 cm × 50 cm × 40 cm arena. Thirty-minute trials were video-recorded by an overhead camera and analyzed using the automated video tracking software ANYmaze 4.9 (Stoelting, Wood Dale, IL, USA). The arena was cleaned with water at the beginning of testing and in between animals. Total distance traveled and time spent in the OF center was measured. For analysis of time spent in the center of the arena, a center zone was virtually defined as a 25 cm × 25 cm central square.

### Forced swim test

Mice were placed in a 2 L glass beaker filled with room-temperature (22 ± 1°C) water to a height of 15 cm so that the mouse could neither touch the bottom nor escape. The test lasted 6 min and was later analyzed by an experienced experimenter, blind to the experimental group. The first 2 min were designated a habituation period, and therefore time spent immobile and time spent struggling in the final 4 min of the test were scored (Castagne et al., 2009).

### Acute stress response

The FST additionally served as an acute stressor to examine the stress response. The stress response was performed as previously described (Wagner et al., 2012; Balsevich et al., 2014; Santarelli et al., 2014). Briefly, at the conclusion of the FST, animals were towel-dried and returned to their home cages to recover. At 30-min (stress response) and 90-min (stress recovery) after the onset of the FST, blood samples were taken by tail cut (Fluttert et al., 2000). The 30-min time point was chosen based on previous data indicating a near maximal response of the HPA axis at this time (Droste et al., 2008). Samples were collected in EDTA-coated microcentrifuge tubes (Kabe Labortechnik, Germany) and kept on ice until all samples were centrifuged at 8000 rpm at 4°C for 15 min. Plasma was collected and stored at −20°C. Plasma corticosterone levels were determined by radioimmunoassay using a commercially available kit (MP Biomedicals Inc.; sensitivity 12.5 ng/ml).

### Tissue collection and processing

On day 22 of the experimental timeline, mice were anesthetized with Isofluorane and then immediately sacrificed by decapitation. Basal trunk blood was collected and subsequently processed (as described above). Brains were removed, snap-frozen, and stored at −80°C until use. Adrenal glands were removed, pruned from fat, and weighed.

### Antidepressant metabolite assessment

Mice brains were weighed and then homogenized in the fivefold volume phosphate buffered saline (PBS), containing “Complete Protease Inhibitor Cocktail Tablets” (Roche, Penzberg, Germany) using a Dispomix Drive (Medic Tools AG, Zug, Switzerland).

The blood plasma and the brain homogenates were analyzed using the combined high-performance liquid chromatography/mass spectrometry (HPLC/MS-MS) technique. Analysis was performed using an Agilent 1100 Series (Agilent, Waldbronn, Germany) liquid chromatograph, which was interfaced to the ESI source of an Applied Biosystems API 4000 (ABSciex, Darmstadt, Germany) triple quadrupole mass spectrometer. All samples were prepared using Ostro protein precipitation and phospholipid removal plates (Waters, Eschborn, Germany).

Deuterated CMI (CMI-D3) was used as internal standard. Chromatography was accomplished using an gradient elution in a Accucore RP-MS 2.6 μm column (2.1 × 50 mm, Thermo Scientific, Dreieich, Germany) at a flow rate of 0.3 ml/min and 30°C.

The composition of eluent A was methanol with 10 mM ammonium formate with 0.1% formic acid and water with 10 mM ammonium formate with 0.1% formic acid as eluent B.

The gradient was 0–0.5 min 20% A, 0.5–2 min 20–90% A, 1 min held at 90% A, 3–3.5 min 90–20% A and 3.5–8 min 20% A. The total run time was 8 min and the injection volume was 5 μl.

The retention time for CMI, DCMI and CMI-D3 were 4.9 min, 4.9 min and 4.9 min, respectively. The ion source was operated in the positive mode at 500°C, and multiple reaction monitoring (MRM) collision-induced dissociation (CID) were performed using nitrogen gas as the collision gas. The collision energy was set to 27 V, 33 V and 27 V for CMI, DCMI and CMI-D3, respectively. The transitions monitored during analysis were *m/z* 315 → 86 for CMI, *m/z* 301 → 72 for DCMI and 318 → 89 for CMI-D3. The detection limit for CMI in plasma was 5 ng/ml and 3 ng/g wet weight in brain tissue and 2.5 ng/ml and 3 ng/g wet weight in brain tissue for DCMI.

### Data analysis

All variables were evaluated using IBM SPSS Statistics 18 software (IBM SPSS Statistics, IBM, Chicago, IL, USA). Data were analyzed by 2-way ANOVA for significant effects of treatment and stress. Where the initial test yielded a significant interaction, a Bonferroni *post hoc* test was applied. Where there was only one grouping variable, independent Student’s *T*-test were used. Finally, correlations were analyzed with the Pearson product-moment test. Statistical significance was set at *p* < 0.05. Data are presented as mean ± S.E.M.

## Results

### Body weight and fluid intake

Body weight gain from the onset of CSDS/treatment exposure until sacrifice was not significantly affected by either stress or CMI treatment. There was a significant effect of stress on fluid consumption (stress: *F*_(1,44)_ = 133.195, *p* < 0.001) in which stress significantly increased daily fluid intake. There was however no effect of CMI treatment on fluid intake. Mice exposed to CSDS therefore received a higher dose of CMI compared to control animals receiving CMI treatment (*T*_(16,9)_ = −9.368, *p* < 0.001).

### Behavior and neuroendocrine parameters

**Figure 1:**
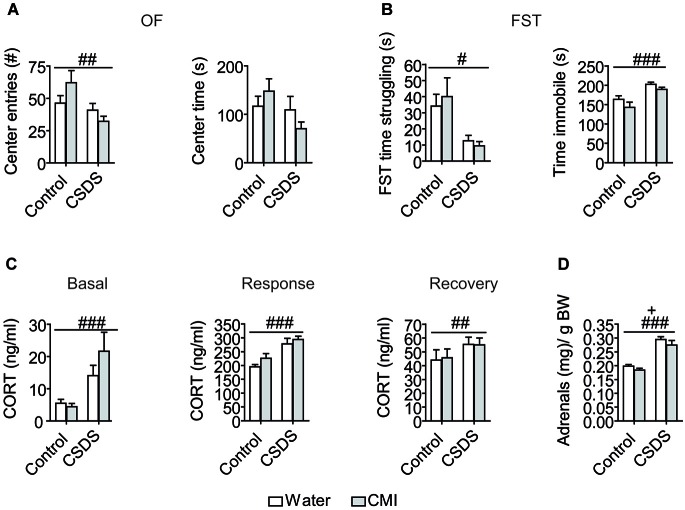
**Effects of CMI treatment and/or chronic defeat stress on behavioral and physiological parameters in mice. (A)** The number of entries into the center and the time spent in the center of a 30-min open field test. **(B)** The time spent struggling and the time spent immobile in the forced swim test. **(C)** Basal morning corticosterone levels as well as response corticosterone levels and recovery corticosterone levels 30 min and 90 min following an acute stressor (FST), respectively. **(D)** Relative adrenal gland weight. All data were analyzed by two-way ANOVA and are represented as the mean +/− S.E.M. ## *p* < 0.01, ### *p* < 0.001, + *p* < 0.05; # significant stress effect and + significant CMI treatment effect.

Stress exposure resulted in increased anxiety-like behavior, as demonstrated by the reduced number of entries into the center (stress: *F*_(1,44)_ = 7.563, *p* = 0.009) and a trend towards reduced center time in the OF test (stress: *F*_(1,44)_ = 3.478, *p* = 0.069). Although there was no significant treatment × stress interaction for either OF center time or center entries, the reduced center time and center entries in the OF test on account of CSDS, appear to be largely mediated by the CMI-treated cohort (Figure 1A). Stress additionally tended to reduce total distance traveled in the OF (stress: *F*_(1,44)_ = 3.024, *p* = 0.089). Likewise heightened depressive-like behaviors resulted from CSDS, shown in the FST as a reduction in time spent struggling (stress: *F*_(1,40)_ = 6.054, *p* = 0.018) and an increase time spent immobile (stress: F_(1,44)_ = 22.211, *p* < 0.001; Figure 1B). Stress furthermore enhanced the stress response as shown by elevated basal (*F*_(1,44)_ = 7.649, *p* = 0.008), response (*F*_(1,44)_ = 25.136, *p* < 0.001), and recovery (*F*_(1,42)_ = 6.590, *p* = 0.014) corticosterone levels (Figure 1C). By contrast, CMI had no main effect on any of the parameters (center time, center entries, total distance) measured in the OF test or on corticosterone levels. There was a trend indicating that CMI treatment reduced the time spent immobile (CMI: *F*_(1,44)_ = 3.413, *p* = 0.071) in the FST (Figure 1B). Finally stress significantly increased adrenal size (stress: (*F*_(1,43)_ = 100.902, *p* < 0.001) whereas CMI treatment led to reduced adrenal size (treatment: (*F*_(1,43)_ = 4.768, *p* = 0.034; Figure 1D).

### CMI and DCMI levels

**Figure 2:**
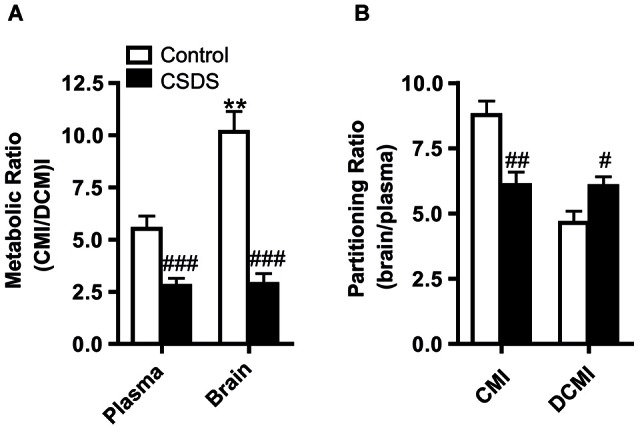
**Stress-induced modulatory effects on CMI pharmacokinetics. (A)** Clomipramine metabolic ratio in the plasma and brain. **(B)** Partitioning ratio for CMI and its active metabolite DCMI. Data were analyzed by Student’s *T*-tests for stress effects and by two-way ANOVA for tissue and stress effects on tissue distribution. All data are represented as the mean +/− S.E.M. **p < 0.01, # *p* < 0.05, ## *p* < 0.01, ### *p* < 0.001; # significant stress effect and * significant tissue effect.

Clomipramine and DCMI levels were measured in the plasma and the brain and the absolute values are presented in Table 1. Briefly, stress exposure significantly lowered absolute levels of CMI in the brain (*T*_(11,7)_ = 2.302, *p* = 0.041), but stress did not significantly affect the levels of CMI in the plasma or DCMI levels in the plasma and brain. Metabolic ratios and partitioning ratios were investigated as an index of CMI pharmacokinetics, thereby also controlling for the observed differences in fluid intake. Assessment of the metabolic ratios (defined as the ratio of CMI to DCMI) revealed an effect of stress. The metabolic ratios were significantly lowered on account of CSDS in the plasma (*T*_(21)_ = 3.981, *p* = 0.001) and in the brain (*T*_(16,41)_ = 6.600, *p* < 0.001; Figure 2A). In order to assess whether tissue distribution of CMI/DCMI was affected by stress, we treated tissue and CSDS as independent variables. Significant effects of stress and tissue were detected (stress: *F*_(1,43)_ = 57.905, *p* < 0.001; tissue: *F*_(1,43)_ = 4.968, *p* = 0.031). Importantly, a significant interaction was also detected to reveal that only under control conditions was the CMI/DCMI ratio significantly higher in the brain compared to the plasma (stress × tissue: *F*_(1,43)_ = 5.343, *p* = 0.026). Regardless, control animals presented a higher CMI/DCMI ratio in both plasma and brain compared to stressed-animals.

**Table 1:** **Absolute concentrations of CMI and DCMI in plasma (ng/ml) and brain (ng/g) tissue**.

Condition	Measurement	Mean +/− S.E.M.	Unit
Control	Absolute CMI plasma	24.2 +/− 5.5	ng/ml
CSDS		14.3 +/− 1.4
Control	Absolute DCMI plasma	4.3 +/− 0.8	ng/ml
CSDS		6.3 +/− 1.3
Control	Absolute CMI brain	224.3 +/− 59.1	ng/g
CSDS		86.2 +/− 10.4
Control	Absolute DCMI brain	23.4 +/− 5.6	ng/g
CSDS		39.2 +/− 8.4

*Data are represented as the mean +/− S.E.M*.

To measure brain uptake of CMI and DCMI, the partitioning ratio (defined as the brain-to-plasma concentration) was calculated. Chronic social defeat stress significantly decreased the partitioning ratio for CMI (*T*_(21)_ = 3.663, *p* = 0.001), whereas significantly increased the partitioning ratio for DCMI (*T*_(21)_ = −2.460, *p* = 0.023; Figure 2B).

### Correlations

**Figure 3:**
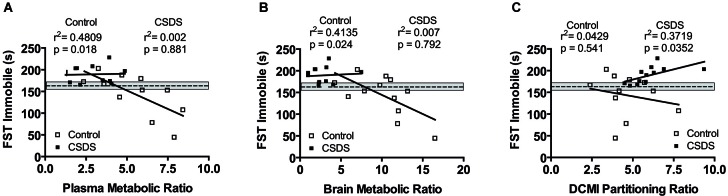
**Correlational analysis between the plasma (A) and brain (B) metabolic ratio and time spent immobile in the FST. (C)** Relationship between DCMI partitioning ratio and time spent immobile in the FST. The mean of the water-treated control group is shown as a dashed line and the corresponding S.E.M. is shaded in gray. Correlations were analyzed with the Pearson product-moment test. Statistical significance was set at *p* < 0.05.

The Pearson product-moment test was used to describe the relationship between CMI bio-distribution and behavioral responses. The analyses were subdivided by stress condition to account for the main stress effects on both CMI bio-distribution and behavioral readouts, which would bias the relationship. Under basal conditions, the CMI/DCMI metabolic ratio for both the plasma and brain negatively correlated to the time spent immobile (floating) in the FST (plasma: *r* = −0.693, *p* = 0.018; brain: *r* = −0.643, *p* = 0.024; Figures 3A,B). This correlation was absent under stress conditions. Finally, the DCMI partitioning ratio positively correlated to the time spent immobile (*r* = 0.610, *p* = 0.035; Figure 3C) under stress, but not under control conditions.

## Discussion

Tricyclic antidepressants, and in particular CMI, are known to display a broad range of inter-individual variation resulting from differences in CMI pharmacokinetics (Shimoda et al., 1995; DUAG, 1999; Vandel et al., 2004). Regardless, the effects of environmental factors, and specifically chronic stress, on CMI metabolism or its distribution remain largely unknown. For this purpose, we chose to examine the effects of stress on CMI bio-distribution and treatment efficacy in an animal model of CSDS, which is accepted as a model for clinical features of depression (Savignac et al., 2011). In agreement with previous studies, CSDS resulted in increased anxiety-like and depressive-like behaviors indicated in the OF and FST, respectively. Stress furthermore resulted in an enhanced stress response. There was however no improvement in anxiety-like behaviors, depressive-like behaviors, or stress reactivity on account of CMI treatment *per se*. Nevertheless stress significantly altered CMI bio-distribution under basal conditions, which in turn predicted anxiety-like and depressive-like behaviors.

Variations in antidepressant pharmacokinetics and serum drug concentrations are considered to be an important reflection of patient treatment outcomes (Noguchi et al., 1993; DUAG, 1999). In order to control for differences in dosage between stress conditions, we evaluated relative values, namely metabolic ratios and partitioning ratios, rather than absolute values. Interestingly, although the mice in the stress group consumed more CMI compared to control animals, the absolute values of CMI assessed in the plasma and brain were similar or even lower in the mice that underwent CSDS compared to controls. The same is true for absolute values of DCMI. Therefore CSDS led to lower absolute brain CMI levels despite an increased CMI dosage.

In order to establish whether stress was able to modulate CMI efficacy, we examined CMI and DCMI levels and bio-distribution. The data suggest that chronic stress affects overall drug levels in the brain and the periphery by promoting extensive metabolism of CMI to DCMI. Interestingly, stress exposure appeared to have a stronger effect in the brain compared to the periphery. For example, under control conditions, the metabolic ratio was significantly higher in the brain compared to the plasma, whereas stress exposure led to comparable brain and plasma metabolic ratios. In clinical practice, therapeutic drug monitoring (TDM) is performed for optimal individualized antidepressant drug therapy in order to adjust for variations in drug absorption, metabolism, and elimination. Of course, TDM is performed on plasma or serum samples, and consequently practitioners are blind to drug concentrations in the brain. Previous findings showed that steady state concentrations of CMI and DCMI strongly reflect brain concentrations in rats, and support the use of TDM for dose optimization (Weigmann et al., 2000). Stress exposure may thus be an important factor to consider for dose optimization as it modulates CMI drug metabolism as well as the relationship between plasma and brain levels. In this regard, life history assessments in psychiatric practice may be a critical prerequisite for individualized intervention.

We further investigated the effects of chronic stress exposure on the drug distribution for CMI and DCMI by evaluating the partitioning ratio. Our data agree with previous findings indicating that the levels of CMI and DCMI are significantly greater in the brain compared to the plasma (Nagy, 1977; Kurata et al., 1986; Fujita et al., 1991; Sgaragli et al., 1995; Weigmann et al., 2000). Interestingly, CSDS significantly lowered the relative amount of CMI in the brain, whereas significantly increased the relative amount of DCMI. The differential effect of stress exposure on the partitioning ratio for CMI and DCMI suggests that DCMI is preferentially sequestered in the brain on account of stress exposure, which may further influence the differential efficacy of CMI treatment.

Next, we explored whether the differential metabolic and partitioning ratios are able to predict the behavioral and endocrine treatment response. We performed the analyses separately for control and stress conditions, as otherwise the strong effects of stress on both behavioral responses and CMI bio-distribution would bias the correlation. Our data indicate that the metabolic ratios under control conditions are predictive for depressive-like behavior observed in the FST (Figures 3A,B). Interestingly, this association is lost under stress conditions. The CSDS cohort presented reduced variability within both the behavioral responses as well as the metabolic ratios, highlighting the great extent to which stress is able to impact both depressive-like behavior and CMI metabolism. In this context, the ability to predict behavioral responses by examining plasma or brain metabolic ratios may be rendered obsolete in a situation of chronic stress, whereby stress may produce a ceiling effect. Nevertheless, the observed associations between the metabolic ratios and FST behavioral readouts in control mice suggests that there is in fact a treatment effect, which depends, in part, on CMI metabolism.

The correlational analyses also revealed that the partitioning of DCMI between brain and plasma is an important indicator of treatment efficacy under conditions of chronic stress, which is in sharp contrast to the associations related to the metabolic ratios whereby behavioral responses were exclusively related to CMI metabolism under control conditions. This again has clinical significance as either the metabolic ratio or partitioning ratio may be an indicator of treatment efficacy in control and stress conditions, respectively. The differences may furthermore underlie differences in activity between CMI and DCMI, given CMI is a stronger inhibitor of serotonin reuptake whereas DCMI is a stronger inhibitor of norepinephrine re-uptake (Balant-Gorgia et al., 1991). Further studies are required to concretely assess the relationship between treatment response and CMI metabolism and bio-distribution, and extend the current findings to other classes of antidepressants. We acknowledge that our study design has its limitations. Future studies are required in order to identify the molecular underpinnings governing the effects of stress on CMI pharmacokinetics. Plausible stress-induced mechanisms resulting in such alterations include the ability of stress to modulate the distribution of multi-drug transporter proteins at the blood brain barrier and thus drug entry into the brain (de Klerk et al., 2010) and/or modulate the activity of CYP2D6 (the enzyme responsible for CMI metabolism), and thus drug bioavailability (Daskalopoulos et al., 2012). Regardless, our study clearly shows a strong effect of stress exposure on CMI metabolic ratios and tissue partitioning, and further suggests that this subsequently regulates treatment responsiveness.

## Conclusion

Collectively, our data illustrate that improved behavioral responses are associated with a higher metabolic ratio of CMI to DCMI, which is in agreement with the situation observed in patients (Dencker and Nagy, 1979; Szegedi et al., 1996). Stress modulates CMI metabolic ratios, which has important implications for dose optimization. To our knowledge, this is the first direct demonstration that chronic stress exposure modulates CMI metabolic ratios and tissue partitioning and subsequently treatment efficacy. This may contribute to CMI’s broad interindividual variability and the widespread efficacy of CMI. Our data strongly promote the implementation of TDM and life history assessments as essential steps in clinical practice to guide individualization of medication (Hiemke et al., 2011), which may likewise indirectly reflect the patient’s stress reactivity.

## Conflict of interest statement

The authors declare that the research was conducted in the absence of any commercial or financial relationships that could be construed as a potential conflict of interest.
